# Bias Sensitivity in Diagnostic Decision-Making: Comparing ChatGPT with Residents

**DOI:** 10.1007/s11606-024-09177-9

**Published:** 2024-11-07

**Authors:** Henk G. Schmidt, Jerome I Rotgans, Silvia Mamede

**Affiliations:** 1 Erasmus School of Social and Behavioural Sciences, Erasmus University Rotterdam, Mandeville Building, Room T15-10, P.O. Box 1738, Rotterdam, DR 3000 The Netherlands; 2 Karolinska Institutet, Solna, Sweden; 3 Institute of Medical Education Research Rotterdam, Erasmus Medical Center, Institute of Medical Education Research Rotterdam, Dr. Molewaterplein 40, Na-2418, 3015 GD Rotterdam, The Netherlands

## Abstract

**Background:**

Diagnostic errors, often due to biases in clinical reasoning, significantly affect patient care. While artificial intelligence chatbots like ChatGPT could help mitigate such biases, their potential susceptibility to biases is unknown.

**Methods:**

This study evaluated diagnostic accuracy of ChatGPT against the performance of 265 medical residents in five previously published experiments aimed at inducing bias. The residents worked in several major teaching hospitals in the Netherlands. The biases studied were case-intrinsic (presence of salient distracting findings in the patient history, effects of disruptive patient behaviors) and situational (prior availability of a look-alike patient). ChatGPT’s accuracy in identifying the most-likely diagnosis was measured.

**Results:**

Diagnostic accuracy of residents and ChatGPT was equivalent. For clinical cases involving case-intrinsic bias, both ChatGPT and the residents exhibited a decline in diagnostic accuracy. Residents’ accuracy decreased on average 12%, while the accuracy of ChatGPT 4.0 decreased 21%. Accuracy of ChatGPT 3.5 decreased 9%. These findings suggest that, like human diagnosticians, ChatGPT is sensitive to bias when the biasing information is part of the patient history. When the biasing information was extrinsic to the case in the form of the prior availability of a look-alike case, residents’ accuracy decreased by 15%. By contrast, ChatGPT’s performance was not affected by the biasing information. Chi-square goodness-of-fit tests corroborated these outcomes.

**Conclusions:**

It seems that, while ChatGPT is not sensitive to bias when biasing information is situational, it is sensitive to bias when the biasing information is part of the patient’s disease history. Its utility in diagnostic support has potential, but caution is advised. Future research should enhance AI’s bias detection and mitigation to make it truly useful for diagnostic support.

**Supplementary Information:**

The online version contains supplementary material available at 10.1007/s11606-024-09177-9.

## INTRODUCTION

An accurate diagnosis is probably the most important step in restoring the health of a patient. At the same time, reaching such accurate diagnosis is a fragile process sensitive to all kinds of disturbances. First, the same disease may present itself differently in different patients. Moreover, complaints, signs, and symptoms produced by different diseases show considerable overlap, making diagnostic decision-making complex. Hence, physicians sometimes make mistakes. About 50% of these mistakes are diagnostic errors outright.^1,2^ A report from the National Academy of Medicine has underscored the critical nature of diagnostic errors within health care, identifying them as not only prevalent but also potentially among the most harmful patient safety issues.^3^ An important cause of diagnostic errors are biases. Studies conducted retrospectively have revealed that biases contribute to approximately three-quarters of real-life diagnostic errors.^4^ Lack of appropriate knowledge and sensitivity to irrelevant but prominent contextual cues are major sources of such biased decision-making, highlighting the need to understand and mitigate biases in diagnostic reasoning processes.^5–7^

Given that decision support through generative artificial intelligence is being explored as a potential solution to diagnostic errors,^8–10^ it is important to examine whether AI chatbots like the Chat Generative Pre-Trained Transformer (ChatGPT) are less susceptible to errors caused by biasing information. In this article, we present findings from a study comparing the diagnostic performance of medical residents with that of ChatGPT on the same clinical problems. The resident data are drawn from five previously published experiments that assessed diagnostic accuracy on clinical vignettes designed to induce bias versus vignettes without bias-triggering information. We will focus on comparing how ChatGPT and the residents handle clinical cases that are subject to bias versus those that are not.

ChatGPT, an AI language model developed by OpenAI, is based on the GPT architecture.^11^ It generates human-like text by analyzing context, language nuances, and patterns from its extensive training data. This model assists with various tasks such as answering questions, writing essays, providing explanations, and engaging in conversations. As a Large Language Model (LLM), it predicts the next word in a sequence by analyzing word patterns and frequencies within a vast textual dataset. The training process involves unsupervised learning, allowing the model to make predictions without explicit human feedback.^10^ However, two known issues are relevant to the validity of our study. First, the output of generative AI can be influenced by how the chatbot is prompted, leading some to advocate for “prompt engineering” to achieve optimal results.^12,13^ Second, chatbot outputs may be biased if the training data contains biased information.^14^ We will explore both issues while discussing our findings.

There have been several attempts to compare diagnostic accuracy of ChatGPT with performance of physicians.^15–26^ These studies have compared the performance of ChatGPT with criterion diagnoses established by experts or with diagnoses from only a small group of physicians. The present study, however, differs in two significant ways. First, it compares ChatGPT’s diagnostic performance with that of 265 internal medicine and general practice residents who participated in five previously published experiments. Second, these experiments were specifically designed to investigate the negative impact of biasing factors on clinical reasoning. All the experiments revealed that biasing interventions negatively affected diagnostic accuracy compared to control groups not exposed to such interventions, indicating that the participating residents were highly susceptible to these contextual biases.

The main objective of the present study was therefore to find out the extent to which ChatGPT is *also* sensitive to biasing information, information that is such an important cause of diagnostic errors committed by human diagnosticians. To that end, we compared mean diagnostic performance of the physicians involved in the various conditions of these experiments with performance of ChatGPT 4.0 and its predecessor ChatGPT 3.5.

## METHODS

### Participants

Participants were 202 residents in internal and 63 residents in family medicine. These residents were generally juniors, in training for 1 or 2 years. They participated in five experiments aimed at studying the influence of biasing information on diagnostic performance.^27–31^ More detailed information describing participants, materials, and procedures applied in these experiments can be found in Supplement 1.

### Materials

Forty-two clinical vignettes were presented to the participants of these five experiments. The vignettes described a patient’s disease history and presenting complaints. In addition, results of physical examination and laboratory findings were part of the text rarely exceeding 300 words. Each of these vignettes was based on a real patient and had a confirmed diagnosis. See Supplement 1.

### Procedure

The participants’ task was to provide a most likely diagnosis for these clinical vignettes in an open-ended format. One experiment^29^ examined the biasing effect of salient distracting features (SDFs), information that strongly suggests a particular diagnosis that ultimately turns out to be irrelevant to the patient’s problem. In a within-subjects design, participants were confronted with vignettes that either contained SDFs at the beginning of the text, at the end, or no SDFs. Clinical vignettes containing an SDF were diagnosed significantly poorer than cases without an SDF. The number of SDF-related errors increased significantly depending on condition of the experiment, demonstrating that it was the biasing role of the SDF that caused the mistakes.

A second and a third experiment studied the effect on diagnostic accuracy of disruptive patient behaviors such as aggression, lack of trust, care avoidance, or contempt.^27,31^ Participants were confronted in a within-subjects design with either disruptive or neutral versions of the same patient. Compared with responses to the neutral version, residents made significantly more diagnostic mistakes when confronted with a patient displaying disruptive behaviors.

Two experiments studied the effect of availability bias on diagnostic accuracy.^28,30^ In one experiment,^28^ participants had to judge the accuracy of a given diagnosis for a particular disease before being confronted with clinical cases that looked very much like the initial case but in fact harbored different diseases. The expectation (and finding) was that the initial judgement task would bias participants into providing a similar diagnosis for the look-alike clinical cases. The second study had participants first judge the accuracy and comprehensiveness of a Wikipedia page describing characteristics of a particular disease (e.g., Q-fever) before having them diagnose cases that looked like the one on the Wikipedia page but in fact had a different diagnosis.^30^ It is important to note that in the first three experiments the biasing information was part of the clinical cases presented, either as SDFs or as a description of a neutral versus disruptive patient. The biasing information was here *intrinsic* to the patient history. In the availability bias experiments, the biasing information preceded the actual clinical cases and can therefore be considered *extrinsic* to these cases. This distinction is reflected in the ways we analyzed the results of ChatGPT.

The clinical vignettes used in these experiments were submitted to ChatGPT 4.0 and 3.5. The latter was only included because some contributors to the OpenAI Developer Forum consider version 4 worse than 3.5 (https://community.openai.com/t/chatgpt-4-is-worse-than-3-5/588078). For the vignettes in which the biasing information was intrinsic to the case, the following prompt was submitted to ChatGPT: “Please generate a most-likely diagnosis for each of the following clinical cases. I require diagnoses only, no need to provide explanations,” followed by the relevant vignettes. The primary outcome was whether ChatGPT provided the correct diagnosis. Its performance was scored as either 1 (correct diagnosis present) or 0 (correct diagnosis not present). Subsequently, a mean proportion of accurate diagnoses was computed similar to the mean proportion of accurate diagnoses provided by the human participants.

The vignettes involved in the Mamede et al. (2010) availability bias experiment were preceded by the following prompt: “Today you will first be asked to assess the likelihood of a particular diagnosis for a particular case. It concerns 2 cases. I do not need explanations; I just want you to estimate its likelihood in the form of a percentage. Subsequently you will be asked to diagnose 8 cases. For each case you will be asked to generate the most-likely diagnosis. I require diagnoses only, no need to provide explanations,” followed by the judgement task and the 8 cases to diagnose.

The prompt for the Schmidt et al. (2014) study was: “More and more often, patients consult sites such as Wikipedia, a popular online encyclopedia, to check what kind of disease they might have. It is therefore important that the information relayed via this medium be accurate and comprehensive. You are therefore requested to evaluate the quality of information that laypersons would encounter in a Wikipedia entry. Subsequently you will be asked to diagnose 4 cases. For each case you will be asked to generate the most-likely diagnosis. But first: how accurate and comprehensive is the Wikipedia entry for (depending on treatment group either Q-fever or Legionnaire’s) disease? The entry can be found here: (depending on treatment group a link was provided to the Wikipedia entry for either Q-fever or Legionnaire’s disease),” followed by the 4 cases to diagnose*.* All these prompts were identical to the prompts given to the residents. (ChatGPT has the tendency to provide an explanation even if you do not ask for it. We therefore instructed it explicitly that we would not need an explanation.)

### Statistical Analysis

Since responses of ChatGPT are equivalent to the responses of only one person or measurement unit, and therefore by definition lack within-group variance, almost all the studies comparing diagnostic performance between humans and ChatGPT are strictly descriptive lacking inferential statistics.^16,17,21,22,24–26^ A notable exception is a study by Jarou and colleagues.^20^ Given that ChatGPT uses probability models to generate responses, it does not always answer the same prompt with the same response. Therefore, Jarou et al. presented 50 times the same clinical vignettes to ChatGPT. We have chosen the same approach. This allowed us to compute chi-square goodness-of-fit statistics testing for significance. The resident data were considered the expected data. Under the null hypothesis, the observed ChatGPT data would not be significantly different from the expected data indicating that ChatGPT is equally sensitive to bias. Rejection of the null hypothesis would be a sign that ChatGPT was differently affected by bias, showing either more or less susceptibility to the biasing information than the residents.

## RESULTS

Table 1 presents the results of the analyses involving the experiments in which biasing information intrinsic to the history of the patient was manipulated. Each of the experiments included a condition in which no biasing information was presented.

**Table 1:** Mean Diagnostic Accuracy Proportions for Residents, ChatGPT 4.0, and ChatGPT 3.5 in Experiments in Which Biasing Information *Intrinsic* to the Patient History Was Manipulated

Experiment	Treatment	Number of residents	Residents’ actual performance	ChatGPT 4.0 performance	ChatGPT 3.5 performance
Mamede, 2014^29^	Cases containing **no** salient distracting features	72	0.54	**0.58**	0.50
	Cases containing salient distracting features **at the beginning**	72	0.39	**0.25**	0.33
	Cases containing salient distracting features **at the end**	72	0.44	**0.34**	0.33
Schmidt, 2017^31^	Cases describing a **neutral** patient	63	0.64	**0.67**	0.67
	Cases describing a **disruptive** patient	63	0.54	**0.50**	0.67
Mamede, 2017^27^	Cases describing a **neutral** patient	74	0.51	**0.63**	0.63
	Cases describing a **disruptive** patient	74	0.41	**0.50**	0.50

In the experiments summarized in Table 1, the presence of biasing information in a case caused a significant decrease in diagnostic accuracy among residents. The weighted mean drop in accuracy was 12%. Accuracy of ChatGPT 4.0 decreased by 21% while accuracy of ChatGPT 3.5 decreased 9%. Chi-square tests comparing performance of residents with that of ChatGPT led to the following results. For the 2014 Mamede et al. study^29^: chi-square (2, 72) = 5.47, *p* > .05. For the Schmidt et al. study^31^: chi-square (1, 63) = 0.28, *p* > .05. And for the 2017 Mamede et al. study^27^: chi-square (1, 74) = 3.55, *p* > .05. These findings suggest that, like human diagnosticians, ChatGPT is sensitive to bias when the biasing information is part of the patient history.

Table 2 contains the results of the analyses involving the experiments in which biasing information extrinsic to the patient history was manipulated.

**Table 2:** Mean Diagnostic Accuracy Proportions for Residents, ChatGPT 4.0, and ChatGPT 3.5 in Experiments in Which Biasing Information *Extrinsic* to the Patient History Was Manipulated

Experiment	Treatment	Number of residents	Residents’ actual performance	ChatGPT 4.0 performance	ChatGPT 3.5 performance
Mamede, 2010^28^	**Not subject** to availability bias	18	0.55	**0.75**	0.25
	**Subject** to availability bias	18	0.39	**0.75**	0.25
Schmidt, 2014^30^	**Not subject** to availability bias	19	0.70	**0.50**	--*
	**Subject** to availability bias	19	0.56	**0.50**	--*

*ChatGPT 3.5 did not allow the use of websites as the triggering material used in this experiment

When the biasing information was extrinsic to the case, residents’ accuracy decreased between 14 and 16% with a weighted mean decrease of 15%. By contrast, ChatGPT’s performance was *not* affected by the biasing information. For the Mamede et al. study^28^: chi-square (1, 18) = 7.29, *p* < .05, suggesting that expected and observed data differ significantly. For the Schmidt et al. study^30^: chi-square (1, 19) = 1.21, *p* > .05. The latter result suggests that the null hypothesis could not be rejected despite of the fact that ChatGPT was clearly *not* affected by the biasing information. This was probably due to the small difference between conditions in the residents group.

## DISCUSSION

Diagnostic error is a ubiquitous threat to the quality of health care, ^3,4^ and we have argued that many of such mistakes are the result of bias; physicians are led astray because they are sensitive to contextual information that is irrelevant to the task at hand.^5–7^ Artificial intelligence is often perceived as having the potential to improve on the accuracy of diagnoses because of its access to large numbers of data and its ability to integrate these data into coherent diagnostic decisions.^8–10^

In the present study, we have attempted to assess to what extent ChatGPT, like physicians, is sensitive to biasing information. To that end, we compared performance of ChatGPT with that of residents in five experiments aimed to study the negative effects of biasing information on diagnostic decision-making.^27–31^ We made a distinction between information that was intrinsic to the case and information that was extrinsic to it. Intrinsic biasing information is part of the patient’s disease history, such as the presence of salient distracting features or information about disruptive behaviors of a patient. Extrinsic biasing information is not part of the patient disease history. The example studied in this article was availability bias, the earlier confrontation with a look-alike patient.^28,30^ See for a review of experiments testing effects of intrinsic and extrinsic biases Schmidt et al.^32^

Our findings can be summarized as follows.

First, when biasing information is part of the patient’s disease history or describes disruptive behaviors, ChatGPT, like humans, makes more mistakes than when bias is absent. The question is why this is so. The reader is reminded that the cases used were experimentally altered to trigger the biased diagnoses. For instance, in the Mamede at al. (2014) experiment, the vignette of a patient with stomach cancer was altered by including the following salient distracting features in the two experimental versions: “(the patient is) a smoker with a history of chronic NSAID medication for osteoarthritis and with a father who suffered from a stomach ulcer, as shown by the family history,” leading some residents to conclude that this patient in fact suffered from a peptic ulcer. Since ChatGPT operates upon probabilistic dependencies among concepts, the occurrence of NSAID medication and stomach ulcer in the family might have led to increasing the probability of peptic ulcer as a likely diagnosis and therefore decreasing the probability of stomach cancer. And indeed, such SDF-driven diagnoses appear in the responses of ChatGPT, adding to diagnostic error. Even more surprising is the fact that a mere description of the behaviors of a disruptive patient, in the absence of a hint to a particular disease, is sufficient for ChatGPT to produce diagnostic errors. This suggests that ChatGPT conjectures that these behaviors are *also* a sign of disease and takes them into account.

Second, when bias is situational, such as a look-alike patient seen before, then ChatGPT is *not* sensitive to the biasing information. Since the vignettes themselves were the same under the different conditions of the experiments, we found that ChatGPT clearly only responds to the case as presented, ignoring biasing information in the context. We made several attempts to “nudge” ChatGPT into taking the context into account. In one attempt regarding the Mamede et al. (2010) availability bias experiment, we added to the prompt displayed in the “METHODS” section: “Please consider the 8 cases with the result of the confirmation task in mind.” This did not change ChatGPT’s response. A second attempt consisted of the following additional prompt: “I would like you to indicate whether or not the 8 cases are similar to the 2 cases you have encountered previously.” Even by explicitly being asked whether the criterion cases were similar to the biasing diagnosis, ChatGPT responded by suggesting the unbiased diagnoses as its most likely choice. It seems that only if ChatGPT ponders the biasing information to be an integral part of the case, it computes diagnoses that demonstrate the influence of bias.

Our findings give rise to some further considerations.

ChatGPT’s responses are as good as the materials on which it was trained. If the materials are biased in some way, for instance regarding gender, race, or culture, ChatGPT’s output would tend to reflect this.^14,33^ Since it is not known which clinical case data are included in its training, it is difficult to check whether the mistakes made by ChatGPT in our study derive from errors in the training materials rather than from our experimental manipulations. We have checked our clinical vignettes against internet sources, notably Wikipedia and the National Library of Medicine, but could not find cases containing the same biasing information as applied in our experiments. In addition, our vignettes are not in the public domain. Nevertheless, a possible bias in some of ChatGPT’s training materials puts limits on our findings.

Some authors maintain that ChatGPT’s accuracy is a function of the nature of the prompts used.^12,13^ It is possible that if we would have used prompts different from the ones described in the “METHODS” section ChatGPT would have made fewer mistakes, another possible limitation. However, to be able to directly compare the performance of the residents with ChatGPT, it was mandatory to use in both cases the same prompts. In addition, when we tried to nudge ChatGPT into considering different information while deciding on the most likely diagnoses, its output was not affected.

Our sample consisted solely of junior residents. This is an additional limitation to our findings because these residents may not be representative for the wider population of physicians. However, other studies show that more experienced physicians are also influenced by biasing information.^34,35^ A final limitation is that we presented these residents with textual vignettes rather than more realistic clinical cases. On a different note, clinical vignettes are extensively used in the study of diagnostic error. Peabody and colleagues have demostrated that vignettes are as valid as standardized patients or chart abstraction if one wishes to study the quality of health care decisions.^36^

A final consideration is the following. Like humans, ChatGPT is error prone. Even under conditions in which experimental biasing information is absent, it performs only slightly (if consistently) better than the junior residents involved in these experiments. These findings are in general agreement with other studies involving ChatGPT in diagnostic reasoning.^15–19,21–26,37^ These findings imply that for now, applying ChatGPT to diagnostic problems should only be done under caution. It is fallible, like we are.

## Supplementary Information

Below is the link to the electronic supplementary material.

Supplementary file1 (DOCX 19 KB)
