# Supplementary material for: Bias Sensitivity in Diagnostic Decision-Making: Comparing ChatGPT with Residents
Source: J Gen Intern Med. 2024 Nov 7;40(4):790–5. doi: 10.1007/s11606-024-09177-9 (PMC11914423; doi:10.1007/s11606-024-09177-9)
Supplement: Supplementary file 1 — Supplementary file1 (DOCX 19 KB) [file 11606_2024_9177_MOESM1_ESM.docx]

**Supplement 1**

**To “Bias Sensitivity in Diagnostic Decision-Making: Comparing ChatGPT’s with Physicians’ Performance”**

**Additional information on the methods of the experiments included in the present study**

**Mamede et al., 2014. Why do doctors make mistakes? The role of salient distracting clinical features**

In a 2012 experimental study conducted at Erasmus Medical Center, Rotterdam, 72 internal medicine residents (mean [SD] age, 29.2 [2.6] years, 49 female) diagnosed 12 clinical vignettes (6 simple; 6 complex) in 3 different formats that differed regarding the presence of salient distracting features (SDF). SDF are findings in a case that tend to grab physicians’ attention, because they are strongly associated with a particular disease that seems at first glance a plausible diagnosis but are indeed unrelated to the problem.

Each clinical vignette consisted of a brief description of a patient’s medical history, signs and symptoms, and findings from physical examination and lab tests. Six vignettes were complex, i.e., consisting of diseases that are not frequently seen or atypical presentations of diseases. The other six vignettes were simple, consisting of typical presentations of frequent diseases. We created three versions of each vignettes: without a SDF, with a SDF presented at the beginning of the vignette, and with a SDF presented at the end of the vignette. To prepare the second and the third versions of the vignette, a sentence displaying a salient distracting feature was added to the original version of the vignette, either relatively early or towards the end of the description. In other words, the vignettes were the same in all versions, presenting exactly the same findings, except for the presence and location of a SDF.

In a within-subjects design, each participant diagnosed the 12 vignettes in the three different formats: 2 simple vignettes without SDF, 2 simple vignettes with SDF in the beginning, 2 simple vignettes with SDF at the end, 2 complex vignettes without SDF, 2 complex vignettes with SDF in the beginning, and 2 complex vignettes with SDF in the end. Which vignette were diagnosed in which format was counterbalanced across participants. The participants were requested to provide the diagnosis as free text. After diagnosing the 12 vignettes, participants were requested to assess, for each vignette, how confident they were with their diagnosis, and how much effort they had invested to diagnose the vignette. Time to diagnose each vignette was also registered. The diagnoses provided by each participant were scored by expert internists by following a standard procedure. Main outcome measurements were: diagnostic accuracy scores (range 0 – 1), frequency of diagnoses associated with the SDF, time to diagnose, confidence in the diagnosis and mental effort.

**Schmidt et al., 2017. Do Patients’ Disruptive Behaviours Influence the Accuracy of a Doctor’s Diagnosis? A Randomized Experiment**

The study was a randomized controlled experiment conducted with 63 family practice residents (mean age M = 31.34; standard deviation SD = 4.00; 44 female) from the Erasmus Medical Center, Rotterdam. All residents were in the last quarter of the third year of their training.

Six vignettes were created for the study. In each vignette, a few sentences described aspects of the patient’s behaviour. These sentences portrayed either a difficult patient or a neutral patient, effectively producing two versions of the same clinical vignette. All vignettes had a confirmed diagnosis and consisted of a brief description of a patient’s history, complaints, symptoms, and findings from physical examination and tests. The diagnoses were: (1) community-acquired pneumonia, (2) pulmonary embolism, (3) meningoencephalitis, (4) hyperthyroidism, (5) appendicitis, and (6) acute alcoholic pancreatitis.

Each participant saw three vignettes in the neutral version and three vignettes in the difficult behaviour version. The vignettes were counterbalanced in an incomplete within-subjects design. The physicians were asked to evaluate the vignettes and make the patient’s diagnosis intuitively and then through deliberate reflection. Finally, the participants rated the patient’s likability. In addition, amount of time needed to arrive at a diagnosis was measured. Main outcome measurements were: diagnostic accuracy scores (range 0 – 1); time spent on diagnosis; patient’s likeability.

**Mamede et al., 2017. Why Patients’ Disruptive Behaviours Impair Diagnostic Reasoning: A Randomized Experiment**

The study was a randomized controlled experiment with 74 internal medicine residents (mean age M = 29.35; standard deviation SD = 2.22; 46 female) from the Erasmus Medical Center, Rotterdam, the Netherlands.

Eight written clinical vignettes, prepared by two board-certified internists by adapting cases used in previous studies, were used in the study. Each vignette consisted of a brief description of a patient’s medical history, present complaints, symptoms and findings from physical examination and diagnostic tests. The diagnoses were: Inflammatory bowel disease, acute viral hepatitis, celiac disease, Addison’s disease, liver cirrhosis, aortic dissection, appendicitis, hyperthyroidism. A fragment of text describing the patient’s behaviours either in the present or in previous visits was added to each vignette. The fragment described either a difficult patient’s behaviours or a neutral patient’s behaviours, thereby leading to two versions of each clinical vignette. We developed portrays of patients with the following behaviours: (1) “frequent demander”, (2) an aggressive patient, (3) a patient who questioned his doctor’s competence, (4) a patient who ignores his doctor’s advice, (5) a patient who has low expectations of his doctor’s support, and (6) a patient who presents herself as utterly helpless; (7) a patient who threatens the doctor; (8) a patient who accuses the doctor of discrimination.

The study employed a balanced within-subjects incomplete block design in which each participant diagnosed half of the vignettes in the difficult patient version and the other half in the neutral patient version, but which vignette was diagnosed in each version differed between participants. The vignettes were counterbalanced in such way that, at the level of the group of participants, all the difficult versions and the neutral versions were diagnosed the same number of times. Each participant first diagnosed the eight clinical vignettes (four neutral and four difficult patient cases). After diagnosing each vignette, participants were asked to recall the patient’s clinical findings and behaviours**.** Main outcome measurements were: diagnostic accuracy scores (range 0 – 1); time spent on diagnosis, and amount of information recalled from patients’ clinical findings and behaviours.

**Mamede et al., 2010. Effect of availability bias and reflective reasoning on diagnostic accuracy among internal medicine residents**

Thirty-six out of 42 eligible internal medicine residents from the Erasmus Medical Center, Rotterdam (mean age, 29.5 years; SD, 2.1) in their first (n = 18) or second (n = 18) year of the residency program participated in the experiment. The study consisted of 3 phases conducted sequentially in a single session. *Phase 1*, exposure, required participants to evaluate the accuracy of a diagnosis provided for 6 different clinical vignettes. *Phase 2*, non-analytical diagnosis, required participants to diagnose 8 new clinical vignettes, 4 of which had clinical manifestations that were similar to 2 of the diseases encountered in Phase 1. For example, a patient with cirrhosis or primary sclerosing cholangitis (diagnosed in Phase 2) may present with signs and symptoms similar to acute viral hepatitis (evaluated in Phase 1). This was expected to induce an availability bias for those 4 clinical vignettes and reduce diagnostic accuracy. *Phase 3*, reflective diagnosis*,* required participants to reflect on the diagnosis of the 4 clinical vignettes that could have been influenced by an availability bias in Phase 2. This was expected to overrule the bias and lead to more accurate diagnoses.

In total, 16 written clinical vignettes were used in this study. The clinical vignettes consisted of a brief description of a patient's medical history, signs and symptoms, and tests results. All vignettes were based on real patients with a confirmed diagnosis. They were prepared by experts in internal medicine and used in previous studies with internal medicine residents. The participants provided the diagnosis as free text, and their responses were evaluated by expert internists by following a standard procedure.

Main Outcome Measures. Diagnostic accuracy scores (perfect = 4.0) on vignettes solved with or without previous exposure to similar problems, through non-analytical (Phase 2) or reflective reasoning (Phase 3), and the average number of times a biased (i.e., Phase 1) diagnosis was given.

**Schmidt et al., 2014. Exposure to media information about a disease causes doctors to misdiagnose similar-looking clinical cases**

The participants of the study were 38 physicians in training to become a specialist in internal medicine (mean age, 29.0 years, S*D,* 2.25 years; 23 female) from 4 university teaching hospitals in the Netherlands. The experiment consisted of three phases. In the first phase, the physicians were requested to read and evaluate the quality of the Wikipedia entry about one of two diseases (Q-fever or Legionnaire’s disease). Six hours later, in the second phase, which was presented as an unrelated study, they diagnosed eight clinical vignettes; two vignettes superficially resembled the disease in the Wikipedia entry they had read (bias-expected), two vignettes resembled the other disease they had not read about (bias-not-expected). In Phase 3, they diagnosed the bias-expected vignettes again using reflective reasoning. The participants provided the diagnosis as free text, and their responses were evaluated by expert internists by following a standard procedure.

Each vignette consisted of a written description of a patient’s medical history, signs and symptoms, and tests results. Four of the 8 eight vignettes were neutral to the purpose of the experiment (so-called “filler” vignettes), and 4 were test vignettes. Two of those 4 were descriptions of diseases that had clinical manifestations similar to those frequently encountered in patients with Legionnaires’ disease, and 2 were diseases similar to Q fever, though all vignettes had different diagnoses. For example, a patient with viral respiratory infection may present with signs and symptoms similar to Q fever. The outcome measurement was diagnostic accuracy score (range 0 – 1).
